# Identifying immune checkpoints on dysregulated T-cells as prognostic biomarkers for multiple myeloma patients with COVID-19

**DOI:** 10.3389/fimmu.2024.1448653

**Published:** 2024-09-17

**Authors:** Ziping Li, Huiwen He, Fujing Zhang, Haolong Li, Xianghong Jin, Yuhang Song, Shuangjiao Liu, Xuan Wang, Junling Zhuang

**Affiliations:** ^1^ Department of Hematology, Peking Union Medical College Hospital, Chinese Academy of Medical Sciences, Beijing, China; ^2^ Department of Medical Laboratory, Peking Union Medical College Hospital, Chinese Academy of Medical Sciences, Beijing, China

**Keywords:** multiple myeloma, COVID-19, T cells, immune checkpoints, immunotherapy

## Abstract

**Background:**

Broad T cell phenotypic alterations and potential dysfunctions were prominent in COVID-19. There are few and inconclusive data about the role of immune checkpoints for T cell exhaustion/activation during SARS-CoV-2 infection in multiple myeloma (MM) patients.

**Methods:**

We tested T cell subsets and immune checkpoints in 177 MM patients with COVID-19, as well as in 32 healthy infected controls and 42 uninfected MM patients. The percentage of CD4+ and CD8+ subpopulation and immune checkpoints (PD-1, TIGIT, TIM-3, LAG-3, CTLA-4, OX40, and 4-1BB) were evaluated by flow cytometry.

**Results:**

We have found that pronounced lymphopenia and inverted CD4/CD8 ratio in severe COVID-19 patients were especially developed within the first month after infection. And T cell subset dysregulation was persistent in severe patients recovering from SARS-CoV-2 infection. Immune checkpoints on CD4+ T cells were variable and uncorrelated with the level of adaptive immunity, while the proportion of CD4+ T cells was positively correlated with humoral immune response. PD-1 and TIGIT on CD8+ T cells were significantly elevated in severe patients and sustained for more than 2 months, which was associated with impaired cellular immune function. Moreover, exhausted molecules PD-1 and TIGIT on T cells were reduced in immunotherapy patients.

**Conclusion:**

The prolonged T cell dysregulation after severe SARS-CoV-2 infection highlights the close surveillance from reinfection in MM patients even during convalescence. PD-1 and TIGIT on CD8+ T cells could be important prognostic factors to stratify prognosis in MM patients with COVID-19. Moreover, immunotherapy may downregulate the expression of exhausted checkpoints PD-1 and TIGIT, leading to T cell overactivation and severe COVID-19.

## Introduction

During T-cell activation, the immune system uses different checkpoint pathways to maintain co-inhibitory and co-stimulatory signals (1). As a result, the disturbance of immune checkpoints (ICs) is linked to immune dysregulation. During COVID-19 infection, it has become clear that the dysregulated immune response against SARS-CoV-2 in the early phase is pivotal for disease severity (2). As we know, the immune defense in COVID-19 infection is balanced between the effects of suppression and immune response with overactivity resulting in a systemic inflammatory process. It has been reported that the expression of ICs is one of the most important manifestations besides lymphopenia and inflammatory cytokines in COVID-19, contributing to worse clinical outcomes (3). However, there is still a controversy about the relationship between the upregulation of ICs and T-cell exhaustion/activation.

Several studies about the role of ICs in balancing immunological response to COVID-19 have been published. Sabbatino et al. found that programmed death-1 (PD-1) and its ligand programmed death-ligand 1 (PD-L1), played an important role in innate and adaptive immune response by serving as modulators (4). Several groups have shown NKG2A, TIGIT, and CTLA-4 expression as signs of functional T cell exhaustion (5, 6). The results from Shima Shahbaz also showed significant upregulation of TIM-3, VISTA, 2B4, CD160, PD-1, CD39, TIGIT, and Gal-9 on both T cell subsets in COVID-19 patients. However, the expression of these coinhibitory receptors was not associated with an impaired T-cell phenotype (7). Moreover, studies have reported sustained cellular immune dysregulation in individuals recovering from SARS-CoV-2 infection, indicating a prolonged period of immune dysregulation (8, 9).

Multiple myeloma (MM), the second most common hematological malignancy, is characterized by abnormal clonal plasma cells in the bone marrow (10). MM patients experience a high risk of severe clinical symptoms and mortality after SARS-CoV-2 infection for compromised immunity (11). Meanwhile, the persistent immune dysregulation predisposes them to reinfection with SARS-CoV-2 and other pathogens. Understanding the potential link between patients’ immune dysregulation features and disease severity represents a crucial step against severe viral infections in these immunodeficient populations.

To date, most studies have focused on the clinical risk factors of severe COVID-19 in MM patients and the level of adaptive immune response. However, the underlying mechanisms of the immune dysregulation and duration after SARS-CoV-2 infection in MM patients remain unclear. There are few and inconclusive data about the significance of T-cell exhaustion/activation during SARS-CoV-2 infection, and the role of ICs in MM patients with heterogeneous COVID-19 is not available. In the study, we described the evolution of T cell subsets and their key markers of activation and exhaustion in MM patients during COVID-19 breakthrough in China, which aim to identify potential biomarkers and provide important cues for the molecular mechanism of aberrant immune responses induced by SARS-CoV-2 infection.

## Methods

### Patients and clinical characteristics

This was a prospective, longitudinal study, conducted from December 6th, 2022 to September 30th, 2023. All MM patients and age- and sex-matched HCs were recruited at Peking Union Medical College Hospital, Beijing, China. The inclusion criteria for subjects were: (1) age ≥18 years; (2) definitive diagnosis of MM; (3) confirmed COVID-19 infection. The exclusion criteria were: (1) autoimmune diseases or concurrent active malignancies; (2) present infection of human immunodeficiency virus. A group of uninfected MM patients and infected healthy controls (HCs) were also classified as controls. The inclusion criteria for healthy controls: (1) age and sex were matched to the included MM patients; (2) no diagnosis of MM; (3) Other inclusion and exclusion criteria same as enrolled MM patients. All of these MM patients enrolled in our study were not infected with COVID-19 before the outbreak of Omicron. The severity of COVID-19 was defined according to the diagnostic criteria of “COVID-19 diagnosis and treatment Plan (10th trial edition)” (12). Peripheral blood mononuclear cells (PBMCs) were collected from severe COVID-19 infection (n = 21) and non-severe COVID-19 infection (n = 156) patients at two-time points: 2-4 and 8-10 weeks after infections. For uninfected individuals, PBMC samples were collected when they participated in the study. Demographic data, clinical characteristics, laboratory data, and treatment regimens were collected from medical records. The latest follow-up was till September 30th, 2023.

### Cell preparation

Peripheral whole blood samples were centrifuged to separate the cellular fraction and plasma. The plasma was then carefully removed and stored at -80°C. PBMCs were isolated using Ficoll-Paque Plus density gradients (GE Healthcare Life Sciences, USA) according to the manufacturer’s instructions. Isolated PBMCs were cryopreserved in cell recovery media containing 10% DMSO (Gibco, USA), supplemented with 10% heat-inactivated fetal bovine serum, and stored in liquid nitrogen until used in the assays.

### Flow cytometry analysis

Isolated PBMCs from all samples were analyzed by LSRFortessa flow cytometry (BD Bioscience, USA). Dead cells were excluded using LIVE/DEAD red fluorescent reactive dye (Thermo Fisher Scientific, USA). Then cells were stained with fluorochrome-conjugated antibodies for specific surface markers for 30 min at room temperature in the dark. The labeling consisted of one cytometry panel (**Supplementary Table S1**) to analyze immune cell populations and immune checkpoint expression. All specimens were analyzed in duplicates with a coefficient of variation (CV) <5% by two independent technicians under the inter-laboratory quality control. The experiments were repeated if the results exhibited a CV >5% according to the instructions of BD Bioscience. The data were analyzed by FlowJo software (Version 10, Tree Star, USA). The representative gating strategy followed is shown in **Supplementary Figures S1**–**S3**.

### Neutralization assay and Interferon-γ ELISpot assay

The circulating neutralizing antibodies (NAb) toward SARS‐CoV‐2 Omicron BA.4/5 subvariant was determined by competitive enzyme‐linked immunosorbent assay (ELISA) using the SARS‐CoV‐2 Surrogate Virus Neutralization Test (sVNT) assay (Genscript, Nanjing, China). An inhibition rate of ≥ 30 is regarded to be positive. T-cell response was measured by the IFN-γ release via enzyme-linked-immuno-spot assay (ELISpot, Dakewe Biotech, Shenzhen, China). Spot forming units (SFU)/3×10^5^ PBMCs ≥10 was regarded as positive T-cell response.

### Statistics

Categorical variables were described as frequency and percentages. Normally distributed continuous variables were presented as means (± standard deviation [SD]), whereas non‐normally distributed data were presented as medians with interquartile range (IQR). Proportions for categorical variables were compared using the χ2 test, and the Fisher exact test was used when data were limited. The quantized variables of parameters were tested by t-test. Nonparametric variables were tested by Mann Whitney U test or Kruskal-Wallis test. Variables with p values <0.05 in the univariate analysis were further used for multivariate logistic regression analysis. The Spearman rank correlation coefficient was used for correlation analysis. All statistical analysis was performed using SPSS v.27.0 (IBM Corp., Armonk, NY, USA). In all analyses, adjusted p-values were calculated and reported to control for multiple comparisons. P-values less than 0.05 were considered statistically significant.

### Study approval

Written informed consent was obtained from all participants. All procedures were performed in accordance with the ethical standards of the Institutional Medical Ethics Committee (No. I-23PJ688) at Peking Union Medical College Hospital.

## Results

### Baseline characteristics of multiple myeloma patients

A total of 219 MM patients and 32 HCs were finally enrolled. The MM cohort was composed of 177 infected patients and 42 uninfected patients. All 32 HCs were infected and vaccinated. At the final follow-up date, among the whole infected patient cohort, 21 (11.9%) MM patients experienced severe COVID-19. The baseline characteristics are listed in **Table 1**. The median age of severe COVID-19 patients was older than non-severe ones (72 [67-77] vs. 65 [57.75-70]). The proportion of patients with severe COVID-19 was higher in males, R/RMM, active MM disease, immunotherapy within 3 months, comorbidities, and unvaccinated. Moreover, compared to non-severe patients, the lymphocytes, hemoglobin, and platelets in severe patients with were lower during the acute phase (p<0.001, p=0.003, p=0.004, respectively). To date, 5 patients with severe disease have died from COVID-19, while no deaths from COVID-19 in non-severe patients.

**Table 1:** Baseline characteristics of multiple myeloma patients.

Characteristics	severe COVID-19	non-severe COVID-19	Uninfected patients
	n=21	n=156	n=42
**Age groups, n (%)**	72 [67-77]	65 [57.75-70]	66.5 [60.5-72.75]
<65	4 (19.0)	73 (46.8)	17 (40.5)
≥65	17 (81.0)	83 (53.2)	25 (59.5)
Gender (M/F), n (%)			
Male (M)	15 (71.4)	86 (55.1)	22 (52.4)
Female (F)	6 (28.6)	70 (44.9)	20 (47.6)
Immunoglobulin subtypes, n (%)			
IgG	9 (42.9)	79 (50.6)	18 (42.9)
IgA	6 (28.6)	30 (19.2)	12 (28.6)
IgD	2 (9.5)	6 (3.9)	2 (4.7)
LC	4 (19.0)	41 (26.3)	10 (23.8)
R-ISS stage, n (%)			
I	3 (14.3)	18 (11.6)	7 (16.6)
II	10 (47.6)	52 (33.3)	18 (42.9)
III	3 (14.3)	28 (17.9)	6 (14.3)
Unknown	5 (23.8)	58 (37.2)	11 (26.2)
**Immunotherapy ≤ 3 months prior, n (%)**	11 (52.4)	25 (16.0)	10 (23.8)
**Auto-HSCT, n (%)**	5 (23.8)	28 (17.9)	13 (31.0)
MM status at COVID-19, n (%)			
Inactive	11 (52.4)	95 (60.9)	28 (66.7)
Active	10 (47.6)	61 (39.1)	14 (33.3)
**R/RMM, n (%)**	11 (52.4)	56 (35.9)	17 (40.5)
**Comorbidities, n (%)**			
0	2 (9.5)	69 (44.2)	12 (28.6)
≥1	19 (90.5)	87(55.8)	30 (71.4)
**COVID-19 vaccination, n (%)**	5(23.8)	78(50)	14 (33.3)
**Absolute Blood counts at 2-4 weeks after COVID-19 infection**			NA
WBC count	4.07 [2.57-8.17]	5.03 [3.46-6.70]	
Neutrophil count*	3.07 [1.70-6.04]	3.07 [2.09-4.35]	
Lymphocyte count*	0.66 [0.34-0.78]	1.17 [0.82-1.65]	
Platelet*	140 [48.50-165.00]	181 [127.00-238.00]	
Hb (mg/dl)	101 [69.00-113.00]	116 [103.00-130.00]	
**Mortality due to COVID-19**	5 (23.8)	0(0.0)	NA

*p<0.05. MM, multiple myeloma; R-ISS, Revised International Staging System; Auto-HSCT, autologous hematopoietic stem cell transplantation; R/RMM, relapsed and refractory multiple myeloma; NA, Not applicable.

### Factors associated with severe COVID-19

Univariate analyses and multivariate logistic regression were performed to identify factors associated with severe COVID-19. Older age (OR 1.098, 95% CI 1.032 to 1.168, p=0.003), immunotherapy (OR 5.764, 95% CI 2.213 to 15.013, p<0.001), comorbidities (OR 7.534, 95% CI 1.697 to 33.461, p=0.008), and non-vaccination (OR 3.242, 95% CI 1.132 to 9.285, p=0.029) were candidate univariate factors, while the only independent risk factor associated with severe COVID-19 was immunotherapy (OR 5.424, 95% CI 1.883 to 15.623, p=0.002) (**Table 2**). Besides, age (OR 1.097, 95% CI 0.956 to 1.185, p=0.066) and comorbidities (OR 3.948, 95% CI 0.829 to 18.811, p=0.085) showed p-values of <0.1 in multivariate analysis, suggesting they are likely significant predictors. Although our study may be underpowered due to the sample size, the observed trends for age and comorbidities remain consistent. Further studies with larger sample sizes are needed to confirm these findings.

**Table 2:** Univariate and multivariate analysis of potential risk factors of severe COVID-19 in MM patients.

Characteristics	Univariate analysis		Multivariate analysis	
	Odds Ratio (95% CI)	P value	Odds Ratio (95% CI)	P value
**Gender (Male)**	2.035 (0.750 - 5.520)	0.163		
**Age≥65**	1.098 (1.032 - 1.168)	**0.003**	1.097 (0.956 - 1.185)	0.066
Immunoglobulin subtypes				
IgG	Reference			
LC	0.856 (0.249 - 2.950)	0.806		
IgA	1.756 (0.576 - 5.355)	0.323		
IgD	2.926 (0.512 - 16.712)	0.227		
R-ISS stage				
I	Reference			
II	1.154 (0.285 - 4.666)	0.841		
III	0.643 (0.117 - 3.541)	0.612		
**R/RMM**	1.964 (0.785 - 4.913)	0.149		
**Auto-ASCT**	1.429 (0.483 - 4.225)	0.519		
**Immunotherapy ≤ 3 months prior**	5.764 (2.213 - 15.013)	**< 0.001**	5.424 (1.883 - 15.623)	**0.002**
**Active MM status at COVID-19**	1.416 (0.567 - 3.534)	0.456		
**Comorbidities**	7.534 (1.697 - 33.461)	**0.008**	3.948 (0.829 - 18.811)	0.085
**Non-vaccination**	3.242 (1.132 - 9.285)	**0.029**	1.911 (0.602 - 6.061)	0.272

MM, multiple myeloma; CI, confidence interval; R-ISS, Revised International Staging System; R/RMM, relapsed and refractory multiple myeloma; Auto-HSCT, autologous hematopoietic stem cell transplantation. Values of p<0.05 in the table are bolded, indicating statistical significance.

### Differential immune T cell subsets in severe and non-severe individuals

**Figure 1:**
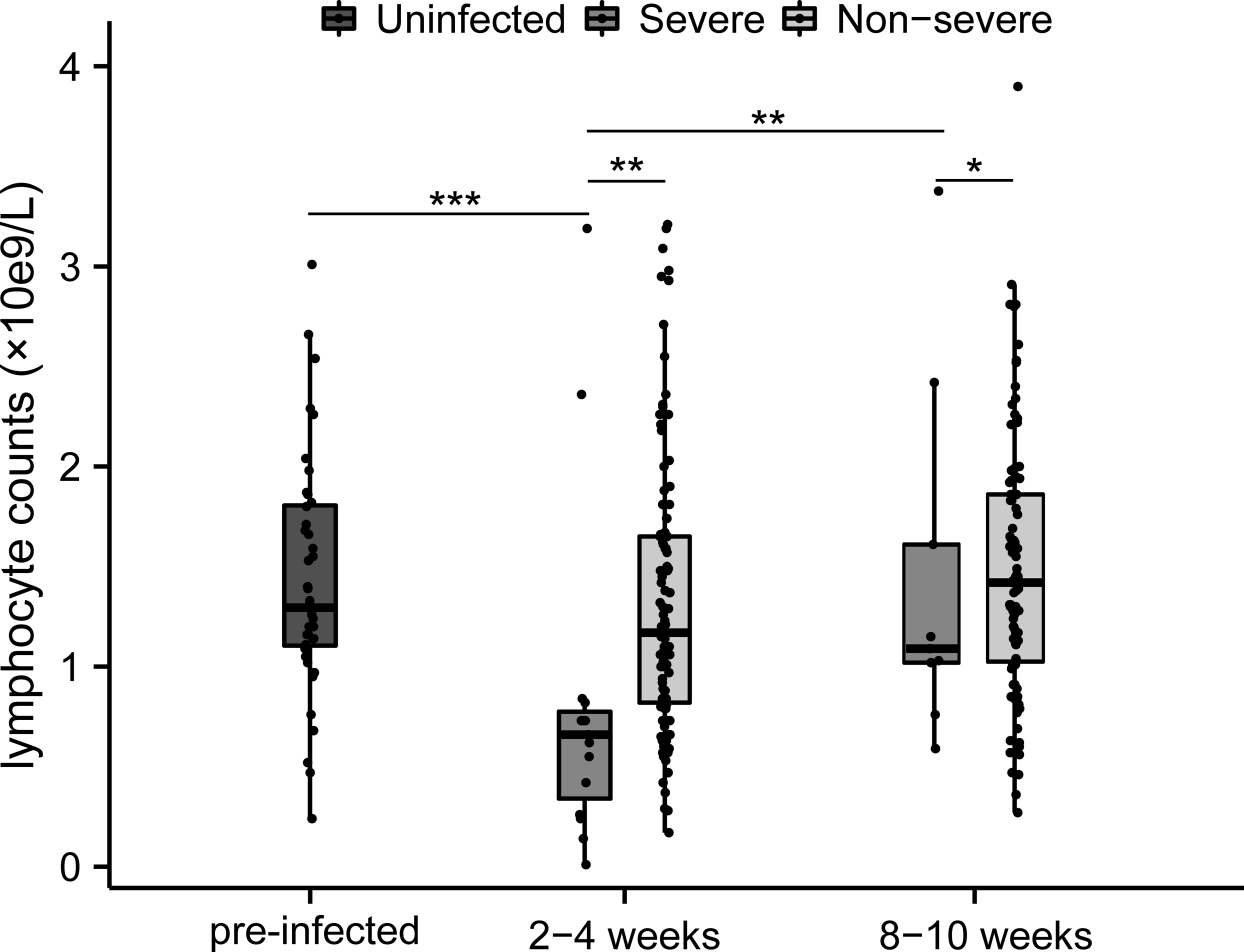
Changes of lymphocytes between different severity groups during COVID-19 infection in MM patients. *p<0.05, **p<0.01, ***p<0.001. The p-values shown are adjusted for multiple comparisons. Median and IQR shown.

**Figure 2:**
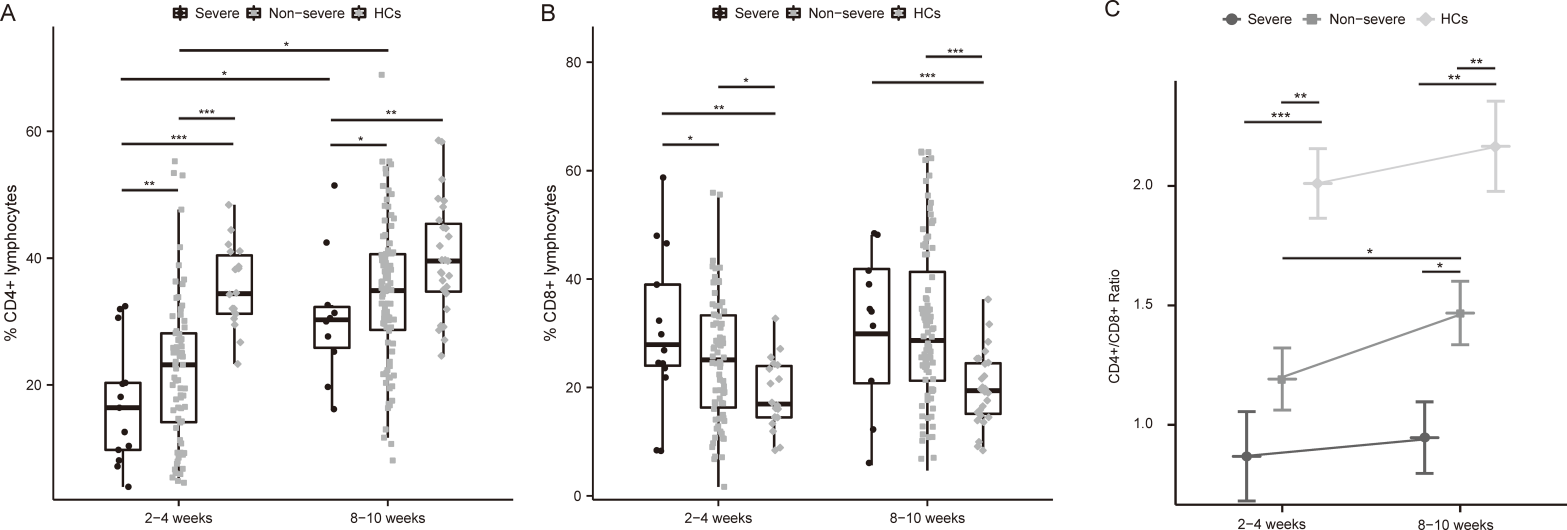
Differential frequencies of T cell subsets and CD4/CD8 ratio in severe and non-severe COVID-19 individuals. **(A)** Dynamics of CD4+ T cell among MM patients with different severity of COVID-19 and HCs after infection. **(B)** Dynamics of CD8+ T cell among MM patients with different severity of COVID-19 and HCs after infection. **(C)** Changes in CD4+ T cells/CD8+ T cells ratio among MM patients with different severity and HCs over time. *p<0.05, **p<0.01, ***p<0.001. The p-values shown are adjusted for multiple comparisons. Median and IQR shown. HCs, healthy controls.

Lymphocyte counts and T-lymphocyte subsets were tested at 2-4 weeks and 8-10 weeks post-infection. Lymphocytopenia was seen in all patients and was particularly pronounced in severe cases (**Figure 1**). The absolute lymphocytes (×10^9^/L) were remarkably lower in severe patients at both time points (2-4 week: 0.66 [0.34-0.78] vs. 1.17 [0.82-1.65], p<0.01; 8-10 week: 1.09 [1.02-1.61] vs. 1.42 [1.03-1.86], p<0.05), which elevated during the recovery of infection (**Figure 1**). Compared to non-severe patients and healthy controls, severe patients represented lower CD4+ T-cells both in the acute phase and convalescence (**Figures 2A, B**). While the proportion of CD8+ T cells was correspondingly higher only at 2-4 weeks post-infection (**Figures 2A, B**). Further, compared with HCs, the CD4/CD8 ratio significantly deviated (reference range: 0.9-2.0) in MM patients at both time points (**Figure 2C**). As the disease progressed towards recovery, the composition of T-cell subsets gradually improved in both severe and non-severe groups, with a significant recovery especially in the non-severe groups (**Figure 2C**). Compared to non-severe patients, severe patients exhibited a lower CD4/CD8 ratio during convalescence (**Figure 2C**).

### The expression of immune checkpoints on CD4+ T cells in severe versus non-severe individuals

**Figure 3:**
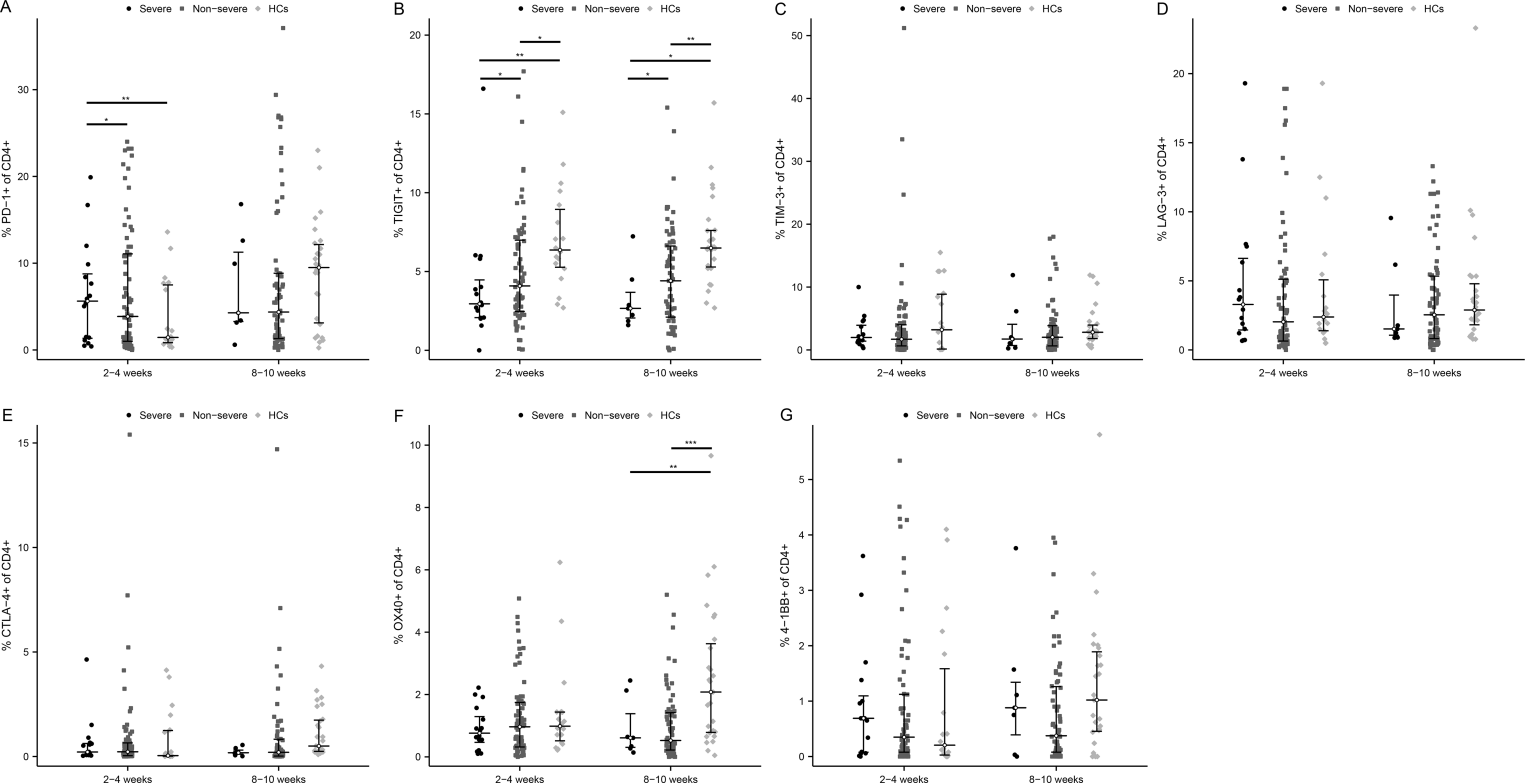
Activated and exhausted immune checkpoints on CD4+ T cell in severe and non-severe COVID-19 individuals. The percentage of each immune checkpoints was evaluated in CD4+ T cells among severe patients, non-severe patients, and HCs at two time points after infection. **(A)** PD-1. **(B)** TIGIT. **(C)** TIM-3. **(D)** LAG-3. **(E)** CTLA-4. **(F)** OX40. **(G)** 4-1BB. *p<0.05, **p<0.01, ***p<0.001. The p-values shown are adjusted for multiple comparisons. Median and IQR shown. HCs, healthy control.

Surface expression of T cell exhaustion markers including PD-1, TIGIT, TIM-3, LAG-3, and CTLA-4, as well as T cell activation markers OX40 and 4-1BB, were tested. Notably, in patients with severe COVID-19, the proportion of PD-1 positive CD4 cells was elevated over those in the non-severe group and HCs at 2-4 weeks (**Figure 3A**, P < 0.05 and P < 0.01, respectively). Interestingly, TIGIT was significantly down-regulated in severe patients at both time points (**Figure 3B**, 2-4 weeks: P < 0.05 and P < 0.01, respectively; 8-10 weeks: P < 0.05 and P < 0.05, respectively). TIM-3 and LAG-3 were also slightly elevated in severe than non-severe individuals at 2-4 weeks (**Figures 3C, D**). While CTLA-4 expression was comparable between the three groups (**Figure 3E**). Further, the activation marker OX40 was lower in MM groups than in HC, especially at 8-10 weeks after infection (**Figure 3F**, p<0.01 in the severe group and p<0.001 in the non-severe group). The level of 4-1BB was low enough (<1%) among the three groups (**Figure 3G**), so it may be difficult to assess its significance in this study.

### The expression of immune checkpoints on CD8+ T cells in severe versus non-severe individuals

**Figure 4:**
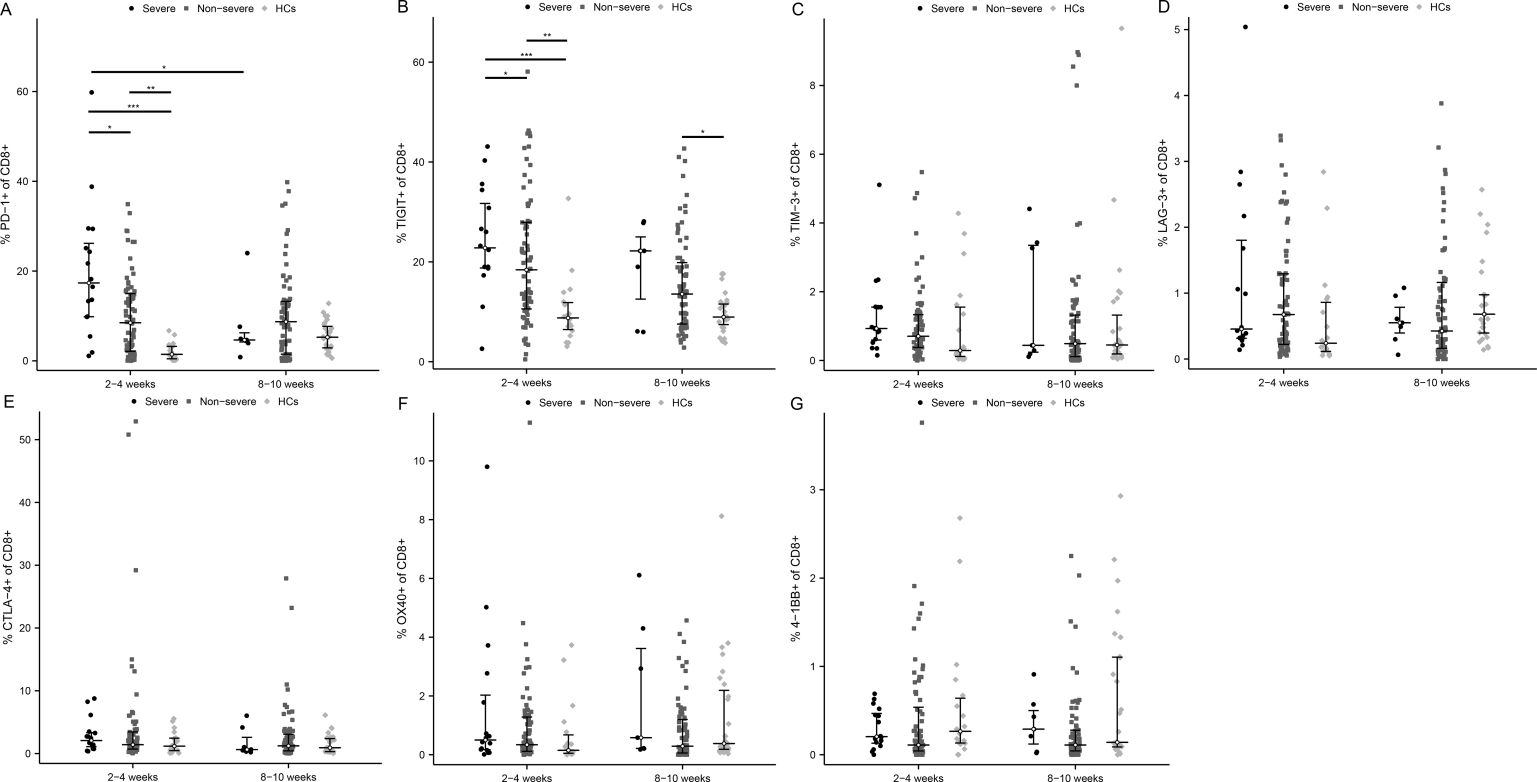
Activated and exhausted immune checkpoints on CD8+ T cell in severe and non-severe COVID-19 individuals. The percentage of each immune checkpoints was evaluated in CD8+ T cells among severe patients, non-severe patients, and HCs at two time points after infection. **(A)** PD-1. **(B)** TIGIT. **(C)** TIM-3. **(D)** LAG-3. **(E)** CTLA-4. **(F)** OX40. **(G)** 4-1BB. *p<0.05, **p<0.01, ***p<0.001. The p-values shown are adjusted for multiple comparisons. Median and IQR shown. HCs, healthy control.

At the acute phase of infection, 2 exhaustion markers were significantly upregulated in severe patients versus non-severe ones and HCs, which were PD-1 (**Figure 4A**, P < 0.05 and P < 0.001, respectively) and TIGIT (**Figure 4B**, P < 0.05 and P < 0.001, respectively). Meanwhile, the positivity rates of PD-1 and TIGIT were significantly higher in non-severe patients compared with HCs (P < 0.01). As COVID-19 improved, PD-1 and TIGIT accordingly diminished in MM patients, especially PD-1 in severe ones (**Figure 4A**, P < 0.01). In addition, TIM-3 and CTLA-4 were also mildly elevated and LAG-3 was decreased early in the infection in critically ill patients (**Figures 4C–E**). Similarly, there were also no significant differences regarding activation markers OX40 and 4-1BB (**Figures 4F, G**).

### T cell subsets and immune checkpoint markers affect adaptive immune response

**Figure 5:**
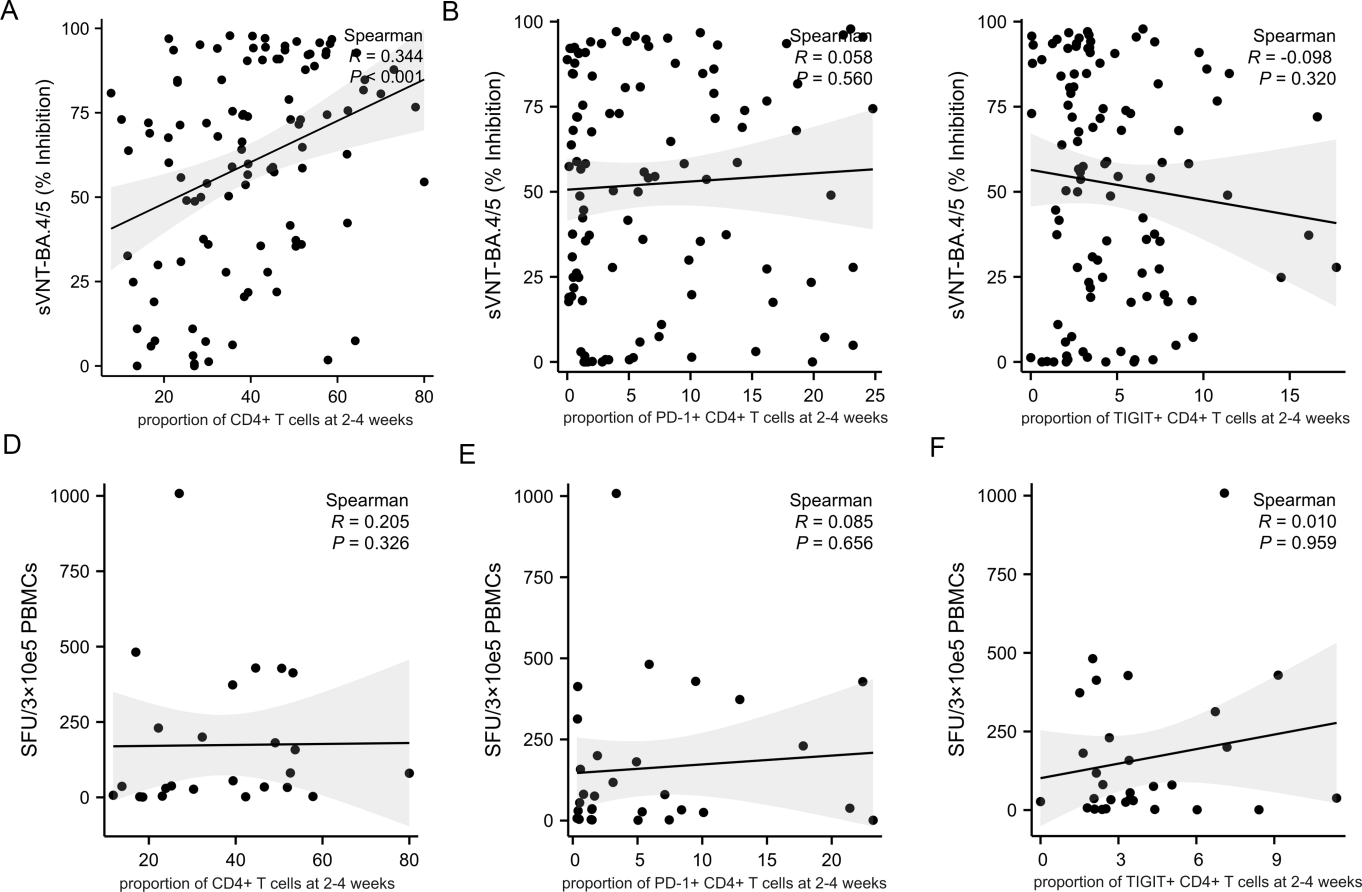
Correlations between CD4+T cell, immune checkpoints frequencies and adaptive immune response in MM patients at 2-4 weeks post-infection. Adaptive humoral immune response: **(A)** CD4+ T cells proportion positively correlates with the level of neutralizing antibody titers. **(B, C)** The frequency of PD-1+CD4+ T cells and TIGIT+CD4+ T cells were not correlate with the level of neutralizing antibody titers. Adaptive cellular immune response: **(D–F)** The frequency of CD4+ T cells, PD-1+ CD4+ T cells, and TIGIT+CD4+ T cells were not correlate with the specific anti-SARS-CoV-2 T-cell response. sVNT, SARS‐CoV‐2 surrogate virus neutralization test; SFU, Spot forming units.

**Figure 6:**
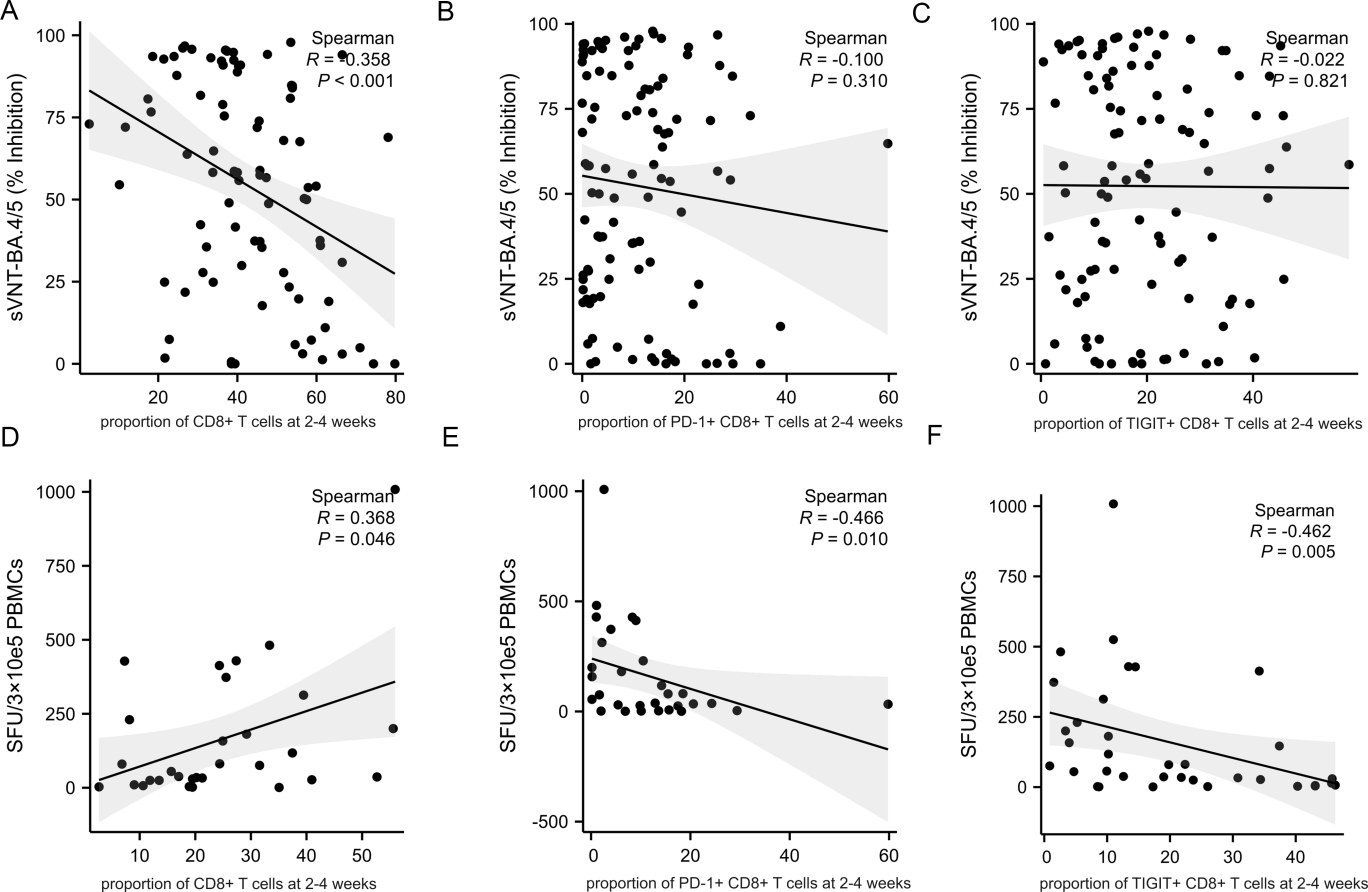
Correlations between CD8+T cell, immune checkpoints frequencies and adaptive immune response in MM patients at 2-4 weeks post-infection. Adaptive humoral immune response: **(A)** CD8+ T cells proportion negatively correlates with the level of neutralizing antibody titers. **(B, C)** The frequency of PD-1+CD8+ T cells and TIGIT+CD8+ T cells were not correlate with the level of neutralizing antibody titers. Adaptive cellular immune response: **(D)** CD8+ T cells proportion positively correlates with the specific anti-SARS-CoV-2 T-cell response. **(E, F)** The frequency of PD-1+CD8+ T cells and TIGIT+CD8+ T cells negatively correlate with the specific anti-SARS-CoV-2 T-cell response. sVNT, SARS‐CoV‐2 surrogate virus neutralization test; SFU, Spot forming units.

As expected, the proportion of CD4+ T cells at 2-4 weeks post-infection positively correlate with the level of neutralizing antibody titers (**Figure 5A**, R=0.344, p<0.001). However, there was no correlation between the proportion of CD4+ T cells at 2-4 weeks post-infection and the cellular immune response (**Figure 5D**, R=0.205, p<0.326). The expression of PD-1 and TIGIT on CD4+ T cells at 2-4 weeks post-infection were variable and uncorrelated with adaptive immunity (**Figures 5B, C, E, F**). The percentage of CD8+ T cells at 2-4 weeks post-infection was reversely correlated with the level of neutralizing antibody titers (**Figure 6A**, R=-0.358, p<0.001). PD-1 and TIGIT expression on CD8+ T cells at 2-4 weeks post-infection did not affect humoral immunity (**Figures 6B, C**). Notably, the proportion of CD8+ T cells at 2-4 weeks post-infection was positively correlated with SARS-CoV-2-specific T-cell response (**Figure 6D**, R=0.368, p=0.046). Significantly, the expression of PD-1 and TIGIT on CD8+ T cells at 2-4 weeks post-infection were negatively correlated with the level of SARS-CoV-2-specific T-cell response (**Figures 6E, F**. R=-0.466, p=0.010; R=-0.462, p=0.005, respectively).

### Effect of immunotherapy on the expression of immune checkpoints

As described previously, immunotherapy is the factor independently associated with severe COVID-19. We further conducted a phenotypic analysis of T cells in uninfected MM patients between the immunotherapy and non-immunotherapy groups. Compared with non-immunotherapy patients, patients receiving immunotherapy had lower expression levels of PD-1 and TIGIT on both CD4+ T cells and CD8+ T cells, although there was no statistical difference (**Supplementary Figure S4**). The expression levels of other immune checkpoints were too low and not significant (**Supplementary Figure S4**).

## Discussion

Here, by analyzing blood samples from both MM patients and healthy controls after COVID-19 infection, we provide a comprehensive overview of alterations of T cell subsets and their key markers for activation and exhaustion. The aims include identifying potential biomarkers for COVID-19 prognosis and providing important cues for the molecular mechanisms of immune dysregulation in MM patients infected with COVID-19.

In the study, we have found that pronounced lymphopenia and inverted CD4/CD8 ratio in severe COVID-19 patients were especially developed within the first month after infection. Further, T cell subset dysregulation was persistent in severe patients recovering from SARS-CoV-2 infection. While the proportion of CD4+ T cells was positively correlated with the function of humoral immunity, the expression of immune checkpoints (ICs) on CD4+ T cells was variable and uncorrelated with adaptive immunity, suggesting that CD4+ T cells may not regulate humoral immunity through selected immune checkpoints pathway. As far as exhausted markers on CD8+ T cells were concerned, PD-1 and TIGIT were even upregulated for more than 2 months in severe patients, which was significantly associated with impaired cellular immune function. Moreover, the expression of exhausted molecules on T cells was suppressed in patients treated with immunotherapy, indicating these T cells may be in a state of hyperactivation prior to infection and correlated with cytokine storms.

Fendler et al. found that the proportion of T cell subsets was unbalanced in tumor patients during the acute infection phase of COVID-19, and the percentages of CD4+ T cells and CD8+ T cells in hematologic tumors were 51% and 42% (13). However, there was few data to evaluate longitudinal changes of T lymphocyte subsets in MM patients after SARS-CoV-2 infection. Our prospective study showed that, both in the acute infection phase and convalescence, the CD4/CD8 ratio was in a state of marked inversion in severe COVID-19 patients. Notably, neither lymphopenia nor T cells subsets dysregulation could return to baseline levels at 2 months post-infection in severe COVID-19 patients. Therefore, we should be vigilant with re-infections and secondary infections in immunodeficient MM patients.

Prior findings reported that, in COVID-19, especially in severe patients, activation and exhaustion markers on the T cell surface were upregulated during acute infection, such as CD69, OX40, CD38, 4-1BB, PD-1, CTLA-4, LAG-3, TIM-3, and TIGIT (4, 14). It has also been found that the upregulation of T cell exhaustion molecules and the downregulation of activation molecules are conducive to the balance of cellular and humoral immune responses and the prevention of deterioration in severe COVID-19 patients (15). Not consistent with previous studies, although the expression of PD-1 on CD4+ T cells was elevated in myeloma patients with severe COVID-19, the expression of TIGIT was instead decreased during the acute phase. Also, the expression of PD-1 and TIGIT on CD4+ T cells did not correlate with the level of humoral and cellular response against COVID-19. This suggested that the changes in exhaustion checkpoints on CD4 + T cells were not uniform and highly variable. Further studies are needed to clarify the specific checkpoint regulating CD4 + T cell function and anti-SARS-CoV-2 humoral response.

The key feature of CD8+ T cell exhaustion is the loss of effector function with sustained expression of inhibitory receptors. A number of studies have reported an exhausted phenotype of CD8+ T cells in severe COVID-19, with increased expression of inhibitory receptors, particularly PD-1 (16, 17). However, Min-Seok Rha et al. demonstrated that PD-1+ CD8+ T cells were not dysfunctional in the acute or convalescent phase of COVID-19, and more likely reflect activation (18). It remains controversial whether CD8+ T cells truly become exhausted when exhausted markers are upregulated.

In the acute infection phase, we found the frequency of PD-1 and TIGIT on CD8+ T cells was significantly higher in the severe COVID-19 cases, which was negatively correlated with cellular immune response. These results indicated that in myeloma patients, the high expression of PD-1 and TIGIT caused impaired CD8+ T cells function, suggesting that the exhaustion of CD8+ T lymphocytes weakened cellular immunity to SARS-CoV-2 in severe patients. The levels of PD-1 and TIGIT might have a prognostic role for MM patients with severe COVID-19.

Combining activation and exhaustion markers on T cells, the expression of related immune checkpoints was persistently dysregulated more than 2 months after SARS-CoV-2 infection. These findings underscore a longer-term influence of immune checkpoint on T cell function in MM patients with COVID-19, particularly those with severe disease.

Immunotherapy is an independent risk factor for severe COVID-19 in MM patients. Defining the immune dysregulation status after immunotherapy would help to account for severe COVID-19. We further analyzed the T cell phenotypes in the immunotherapy versus non-immunotherapy groups of MM patients who had not been infected with COVID-19. The results revealed that both PD-1 and TIGIT were lower in patients receiving immunotherapy compared with non-immunotherapy patients. These findings suggested that immunotherapy could suppress the exhausted molecules and may contribute to T cell overactivation. Previous studies have shown that hyperimmune response during the acute phase could develop a cytokine storm, leading to severe disease (19). Our results implied, but not confirmed, that the anti-MM immunotherapy may cause immune overstimulation by downregulating T cells exhaustion molecules such as PD-1 and TIGIT, associated with more severe COVID-19 in this subgroup.

Importantly, the reduction of T cell counts and T cell dysfunction correlate with a poor prognosis in almost various viral infections. In other words, T cells dysregulation is common in viral infection and may be more severe in immunodeficient populations such as MM. Some immune checkpoint molecules such as PD-1, TIM-3, and TIGIT have been reported to regulate T cells’ destruction during acute and chronic viral infections, including Ebola, HIV, Hantavirus, hepatitis B, dengue virus, and even in the influenza virus (20, 21). Meanwhile, other cancer patients treated with immunotherapy also exhibited poor prognosis when suffering viral infections. All these results suggest that immune checkpoints may play a crucial role in causing severe viral infection by inducing dysfunction of T cells. During the epidemic period of viral infectious diseases, clinicians should be vigilant and adopt effective measures to prevent severe cases in tumor patients who received immunotherapy.

Limitations of our study include a small sample size of immunotherapy patients in uninfected MM patients. Although we did not profile uninfected patients due to sample constraints, which is a limitation of our study, the observed differences of the proportion of T cell subsets between patients and healthy donors could potentially be explained by disease-associated differences at baseline. Our findings still demonstrate a clear difference of the component of T cell subsets between patients who developed severe versus non-severe disease. In addition, the duration of immune dysregulation after infection exceeded our expectations. Combined with confounding factors such as restarting anti-myeloma treatment, secondary infections, comorbidities, etc, we may need longer follow-up to investigate the alteration of immunity sufficiently.

## Conclusions

In conclusion, this study provides broad insight into the regulation of T cells subsets and identifies immune dysregulation in MM patients. The lymphocyte counts and CD4/CD8 ratio were considerably reversed and sustained in patients with severe SARS-CoV-2 infection. It is recommended that high-risk patients should be strictly protected for at least 8-10 weeks to avoid reinfection or secondary infections. PD-1 and TIGIT on CD8+ T cells might contribute to the risk-stratification of COVID-19 patient prognosis. Moreover, immunotherapy may downregulate the expression of exhausted checkpoints such as PD-1 and TIGIT, leading to T-cell overactivation and severe COVID-19.

## Data Availability

The raw data supporting the conclusions of this article will be made available by the authors, without undue reservation.
