# Supplementary material for: Identifying immune checkpoints on dysregulated T-cells as prognostic biomarkers for multiple myeloma patients with COVID-19
Source: Front Immunol. 2024 Sep 17;15:1448653. doi: 10.3389/fimmu.2024.1448653 (PMC11442272; doi:10.3389/fimmu.2024.1448653)
Supplement: Supplementary file 1 [file DataSheet1.pdf]

Table S1: Fluorochrome-antibody conjugates.

Antigen	Fluorophore	Clone	Corporation
CD3	AF700	OKT3	Invitrogen
CD4	SB600	SK3 (SK-3)	Invitrogen
CD8	APC-eF780	RPA-T8	Invitrogen
PD-1	PE-eF610	eBioJ105 (J105)	Invitrogen
TIGIT	PerCP-eF710	MBSA43	Invitrogen
TIM-3	PE	F38-2E2	Invitrogen
LAG-3	SB436	3DS223H	Invitrogen
CTLA-4	PE-cy7	14D3	Invitrogen
OX40	APC	ACT35 (ACT-35)	Invitrogen
4-1BB	FITC	4B4 (4B4-1)	Invitrogen
Live/Dead	aqua		Invitrogen

Figure S1: Immunophenotyping Gating Strategy

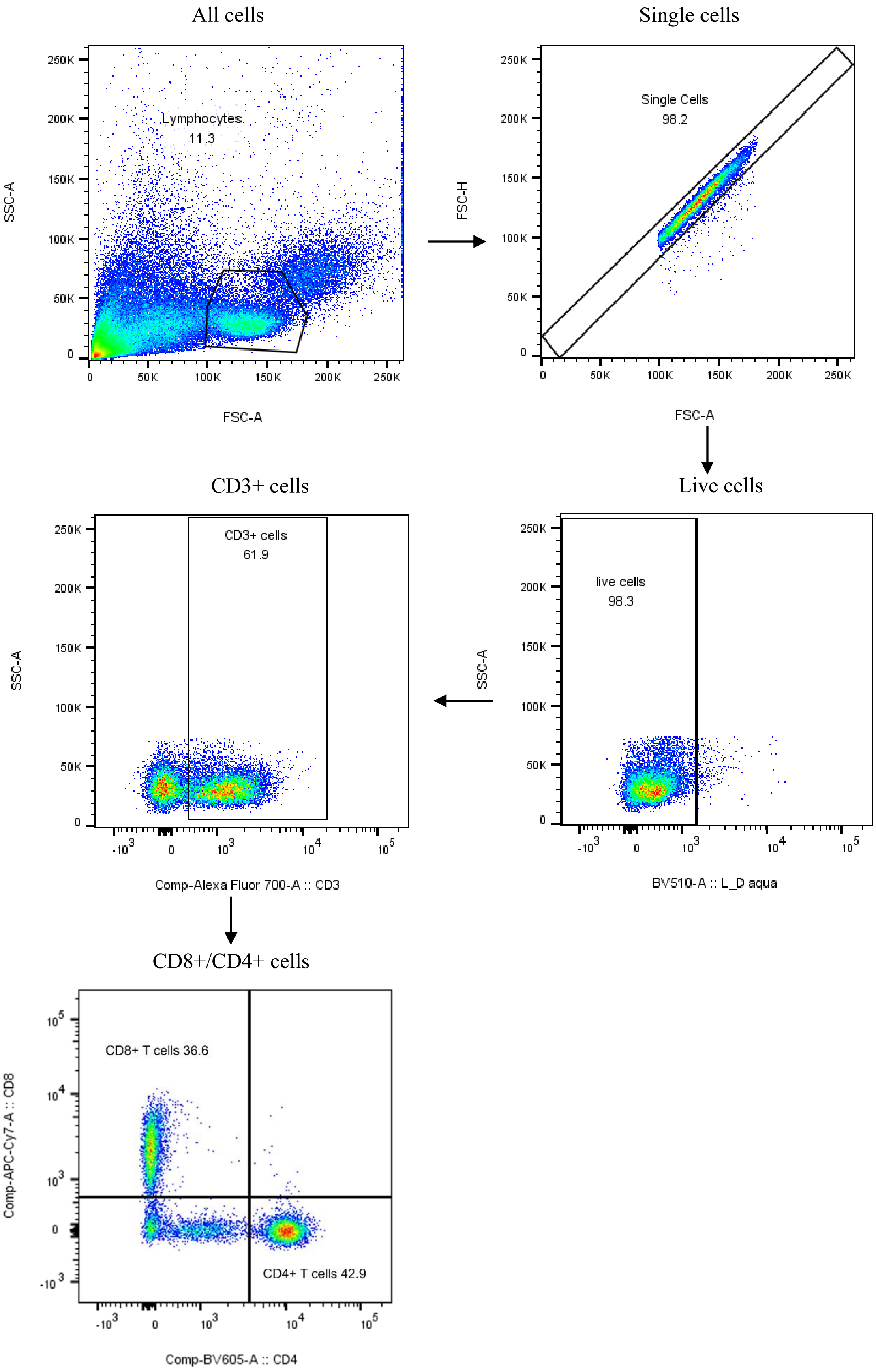

Figure S2. CD4+ T cell panel gating strategy.

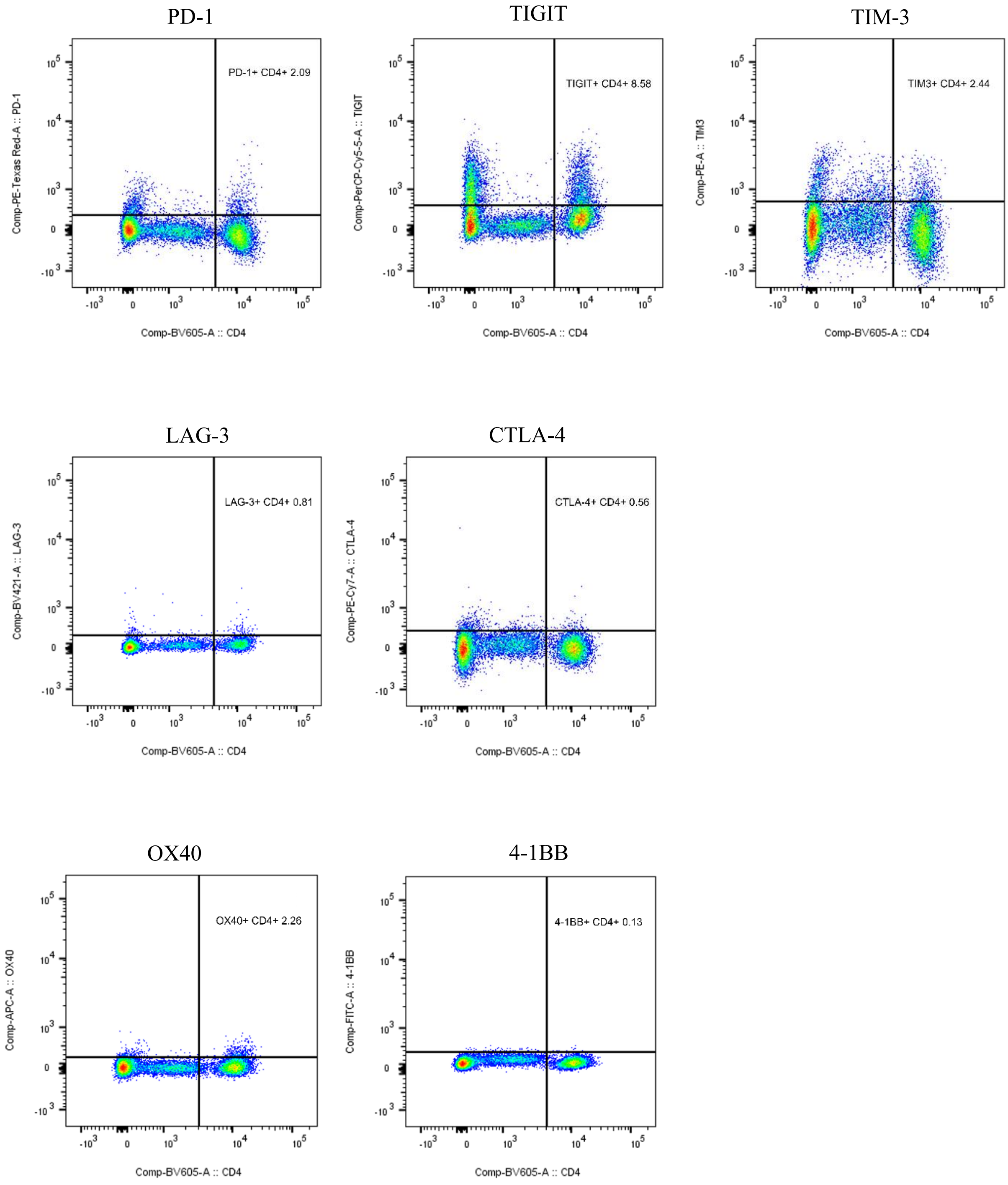

Figure S3. CD8+ T cell panel gating strategy.

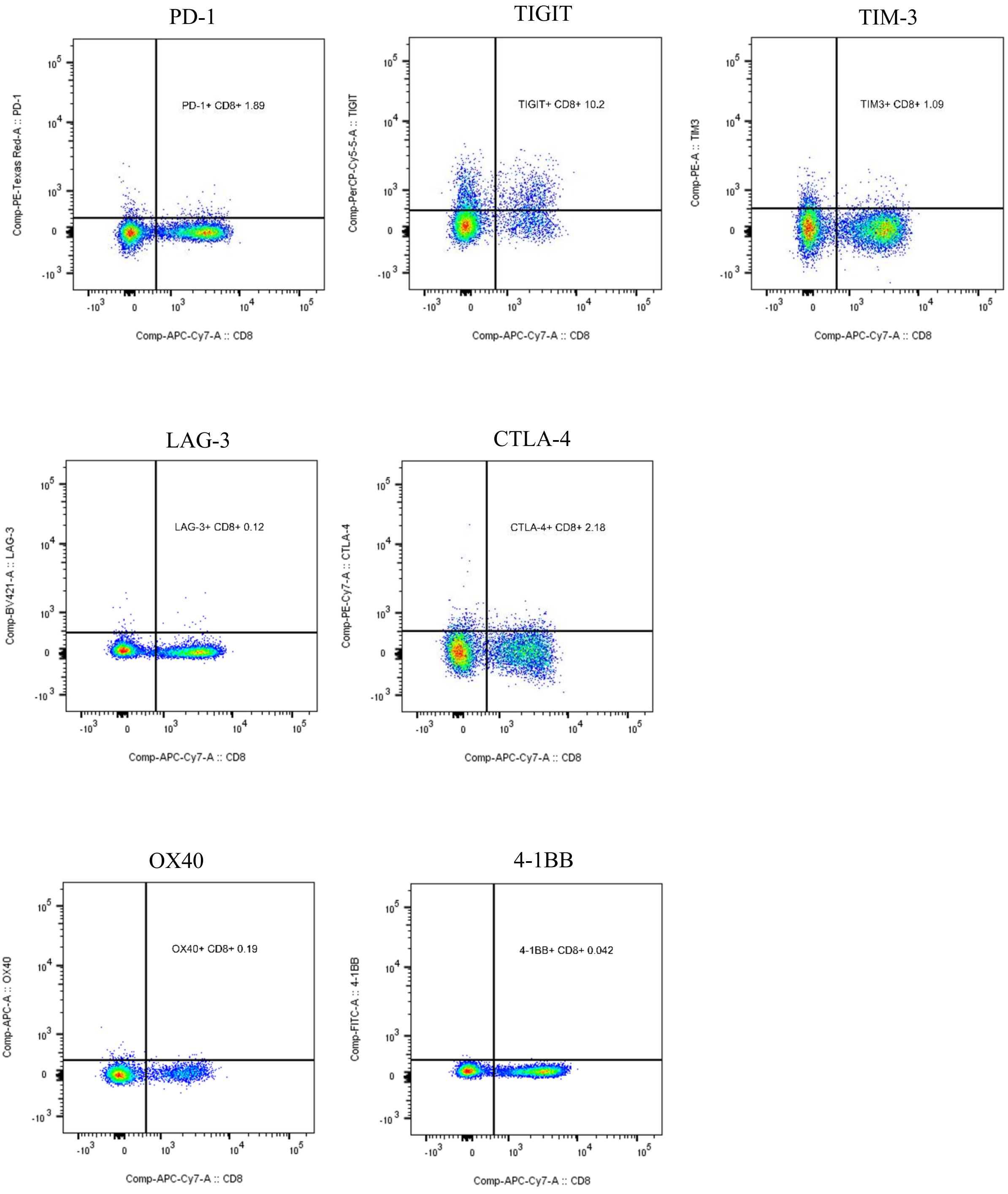

Figure S4. Differential frequencies of immune checkpoints in immunotherapy and non-immunotherapy group.

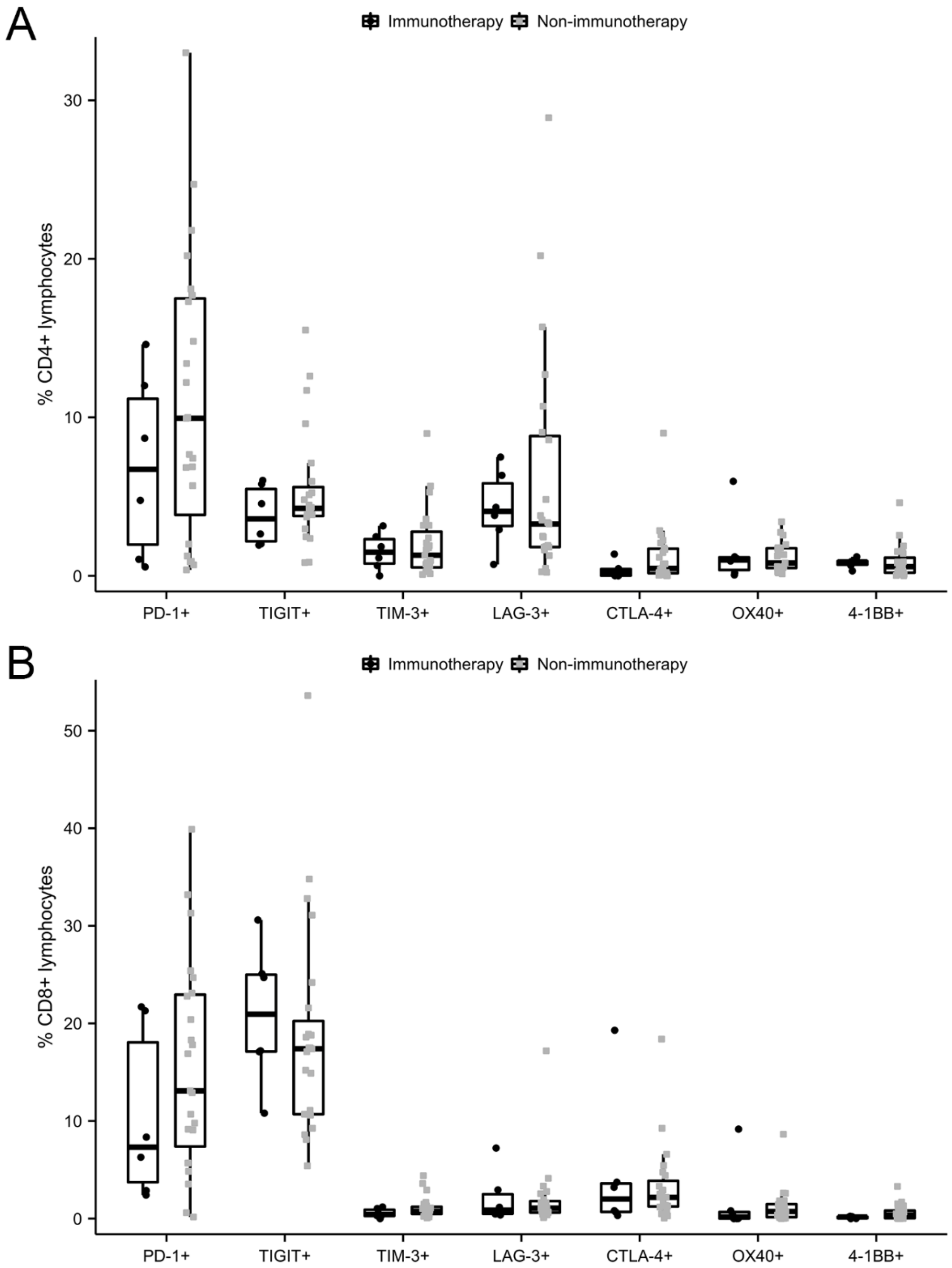

A. The frequency of each immune checkpoint on CD4+ T cells between immunotherapy and non-immunotherapy MM patients. **B.** The frequency of each immune checkpoint on CD8+ T cells between immunotherapy and non-immunotherapy MM patients.
